# Clinical Advances and Perspectives in Targeted Radionuclide Therapy

**DOI:** 10.3390/pharmaceutics15061733

**Published:** 2023-06-14

**Authors:** Nicolas Lepareur, Barthélémy Ramée, Marie Mougin-Degraef, Mickaël Bourgeois

**Affiliations:** 1 Comprehensive Cancer Center Eugène Marquis, 35000 Rennes, France; 2 Inserm, INRAE, Institut NUMECAN (Nutrition, Métabolismes et Cancer)—UMR 1317, Univ Rennes, 35000 Rennes, France; 3 Nuclear Medicine Department, Nantes University Hospital, 44000 Nantes, France; barthelemy.ramee@univ-nantes.fr (B.R.); marie.degraef@univ-nantes.fr (M.M.-D.);; 4 Inserm, CNRS, CRCI2NA (Centre de Recherche en Cancérologie et Immunologie Intégrée Nantes—Angers)—UMR 1307, Université de Nantes, ERL 6001, 44000 Nantes, France; 5 Groupement d’Intérêt Public ARRONAX, 1 Rue Aronnax, 44817 Saint Herblain, France

**Keywords:** radionuclide therapy, nuclear medicine, radiopharmaceutical, antibody, peptide, small molecule inhibitor, tumor, microenvironment

## Abstract

Targeted radionuclide therapy has become increasingly prominent as a nuclear medicine subspecialty. For many decades, treatment with radionuclides has been mainly restricted to the use of iodine-131 in thyroid disorders. Currently, radiopharmaceuticals, consisting of a radionuclide coupled to a vector that binds to a desired biological target with high specificity, are being developed. The objective is to be as selective as possible at the tumor level, while limiting the dose received at the healthy tissue level. In recent years, a better understanding of molecular mechanisms of cancer, as well as the appearance of innovative targeting agents (antibodies, peptides, and small molecules) and the availability of new radioisotopes, have enabled considerable advances in the field of vectorized internal radiotherapy with a better therapeutic efficacy, radiation safety and personalized treatments. For instance, targeting the tumor microenvironment, instead of the cancer cells, now appears particularly attractive. Several radiopharmaceuticals for therapeutic targeting have shown clinical value in several types of tumors and have been or will soon be approved and authorized for clinical use. Following their clinical and commercial success, research in that domain is particularly growing, with the clinical pipeline appearing as a promising target. This review aims to provide an overview of current research on targeting radionuclide therapy.

## 1. Introduction

In the 1940′s, the use of iodine-131 in the treatment of benign and malignant thyroid disorders turned nuclear medicine into a therapeutic reality. Since then, millions of patients, particularly ones with differentiated thyroid carcinoma, have been effectively treated with radioiodine, due to the high affinity of the iodide ion (I^−^) for a transmembrane glycoprotein expressed at the surface of thyroid cells, the sodium/iodide symporter (NIS) [1].

Subsequently, relying on a number of different mechanisms to achieve selective uptake in tissues, other radiopharmaceuticals have appeared for the treatment of various malignant tumors [2,3]. To name a few, small synthetic molecules targeting metabolic processes, such as radiophosphorus and metaiodobenzylguanidine, or targeting extracellular mechanisms, such as bone seeking agents, biological compounds targeting specific cell surface receptors or antigens, or particulates delivered in situ or in the vicinity of the tumor, such as radioiodized oil or radiolabeled microspheres for the radioembolization of liver tumors, or radiocolloids for radiosynovectomy of painful joints in rheumatoid arthritis. Though the concept of using radionuclides as a treatment modality is not new, the latest advances in understanding of the complex molecular mechanisms underlying cancer biology and with the knowledge of radiobiology, nuclear medicine has moved towards the use of more and more specific radiotracers for research and clinical use [4].

**Figure 1:**
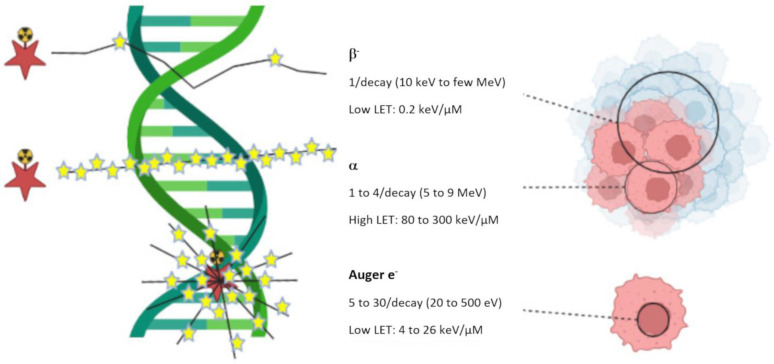
Linear Energy Transfer (LET) for different particle types (β^−^, α and Auger electron). Stars (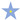) represent disintegrations.

Ionizing radiation causes irreversible damage to the DNA of targeted cancer cells, directly related to the nature and energy of the radiation emitted, which may lead to apoptosis (Figure 1). Unlike external-beam radiotherapy (EBRT), with radionuclide therapy (RNT), the decay of the radionuclide near the target cells delivers a constant radiation dose at a low dose rate (6 versus about 0.01–1.00 Gy/min, respectively). The foremost advantage of RNT over EBRT is the fact that it has proven to be useful for the treatment of both localized tumors and small metastatic tumors spread throughout the body [5].

Radionuclides, radiolabeled molecules, or radiolabeled supramolecular objects (nano- or microparticles) are systemically or loco-regionally injected to target and kill selected cells or tissues while sparing healthy ones. Contrary to molecular imaging, which uses radionuclides that are rather penetrating but not quite ionizing (γ or β^+^-emitting radionuclides), RNT makes use of radionuclides with lower penetrating but more energetic, hence, more ionizing emissions (β^−^, α or Auger e^−^ emitters) (Table 1). As illustrated in Figure 1, β^−^ particles are able to irradiate large volumes of multicellular dimensions. Because of their long range in tissue, β^−^ particles are considered ideal to treat large tumors, but their long range also implies that untargeted neighboring cells may be exposed to irradiation too (cross-fire effect). α particles can irradiate tumors of cell dimensions. Targeted α-therapy (TAT) is therefore generally used for the treatment of small hematological tumors or micrometastases. Auger electrons irradiate volumes with subcellular dimensions. They are especially appropriate for delivering high levels of radiation directly to the nucleus of cancer cells, avoiding off-site damages [6,7]. Main challenge will therefore be the careful targeting to the selected site [8].

There have been impressive results with some therapeutic radiopharmaceuticals or radioactive constructs not actively targeted at a specific site, such as the aforementioned radioiodine, [^223^Ra]radium dichloride, for the management of painful bone metastases in mCRPC patients or radioembolization of liver cancers [1,10,11]. However, the use of a specific vector molecule of a tumor target can allow selective and specific accumulation of radioactivity on its target with a favorable tumor-to-healthy tissue ratio. The ideal target is a receptor overexpressed in a malignant cell, while having no or very little expression in physiological tissues. It should be easily accessible, and thus preferentially expressed on the cell membrane or possess an extracellular moiety [12]. Such targets include cell-surface and transmembrane glycoproteins (such as cluster of differentiation (CD) antigens, folate receptors…), glyco- or phospholipids (e.g., disialoganglioside GD2, phosphatidylserine…), carbohydrates (e.g., lectins), cell-surface receptors (e.g., G protein-coupled receptors), integrins (e.g., α_V_β_3_ …), growth factor receptors (e.g., EGFR and VEGFR), transporters (e.g., LAT1, norepinephrine transporter …), or enzymes (e.g., matrix metalloproteinase …) [13,14]. To address those targets, receptor-specific ligands range from small biomolecules and synthetic inhibitors, peptides and peptidomimetics, mono- and polysaccharides to large proteins and oligonucleotides (antibodies and fragments, aptamers …), peptides and peptidomimetics, mono- and polysaccharides (e.g., mannose, N-acetylgalactosamine, hyaluronic acid…), to small bio- and synthetic molecules (e.g., nucleosides, vitamins, sulfonamides, rucaparib…) [15]. Unlike for targeted therapies and immunotherapy, heterogeneous target expression within the tumor mass is less of a problem for RNT, because it only necessitates a few bound molecules to destroy the target cell. Consequently, it is possible to destroy cells that have low expression levels.

Generally, with β^−^ emitters, it necessitates the specific attachment of several hundred radiotracers to the targeted cell in order to be able to destroy it, whereas α-emitters, due to their high radiotoxicity, only need a few of them. Since several different receptors are likely to be conjointly overexpressed on cancer cells, multitargeting might be an elegant method for enhanced targeting [16].

Over the past two decades, our understanding of cancer biology has improved significantly. Cancer now appears to be a profoundly heterogeneous pathology, with substantial intra- and inter tumoral variability. Thus, the characteristics of cancer have changed from a cell-centered view, with tumors consisting in malignant cells only, to a more comprehensive tissue-oriented one, also including stromal cells and extracellular matrix components, which account for over 90% of the tumor mass and form the tumor microenvironment (TME) [17]. Though varying between patients, there are strong similarities among individuals’ TME phenotypes [18]. The TME is a dynamic ecosystem in which complex interactions occur between malignant cells and the extracellular matrix (ECM), as well as with the resident and recruited cells. It stimulates tumor growth, immune evasion, and metastasis by creating a tumor-permissive microenvironment, characterized by high interstitial pressure, hypoxia, acidosis, angiogenesis and a deregulated metabolism. [19]. It thus plays an essential role in therapeutic resistance [20,21]. In view of these characteristics and their universal expression in a large subset of cancers, TME biomarkers represent attractive targets for RNT [22].

**Figure 2:**
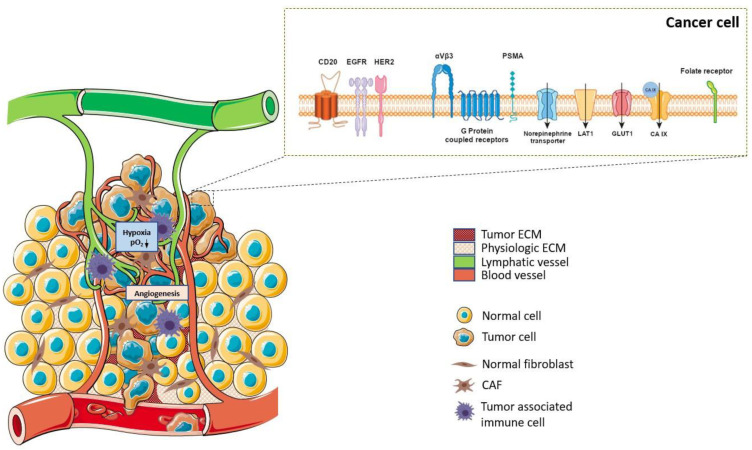
Possible targets for targeted radionuclide therapy.

In particular, cancer-associated fibroblasts (CAFs), have been shown to participate in tumor progression by secreting cytokines, growth factors and exosomes and to inhibit therapeutic response by promoting fibrosis and solid stress [23]. Due to their direct participation in the invasion of neighboring tissues and the resistance to treatment, it has been postulated that selectively irradiating CAFs could lower the risk of recurrence due to residual disease. The immune response has also recently attracted attention as a target of choice [24]. While tumor-associated macrophages (TAMs) have only been targeted with diagnostic radiopharmaceuticals, tumor-infiltrating lymphocytes (TILs) represent one of the currently most promising targets both for imaging and therapy [25,26]. Another strategy could be to target the tumor’s abnormal vasculature, neoangiogenesis, to starve the tumor and eventually kill it [27]. Targeting extracellular matrix and altered TME processes might be another alternative [28]. In solid tumors, microvasculature is often anomalous, resulting in an imbalance between oxygen supply and consumption. As a result, hypoxia is a key feature in most solid tumors and is often associated with poor outcome. In fact, it is closely associated with tumor growth, malignant progression and resistance to chemotherapy and radiotherapy. This could make hypoxia an attractive therapeutic target [29]. All these advances stimulated the exploration of new potential specific targets for RNT [30] (Figure 2).

**Figure 3:**
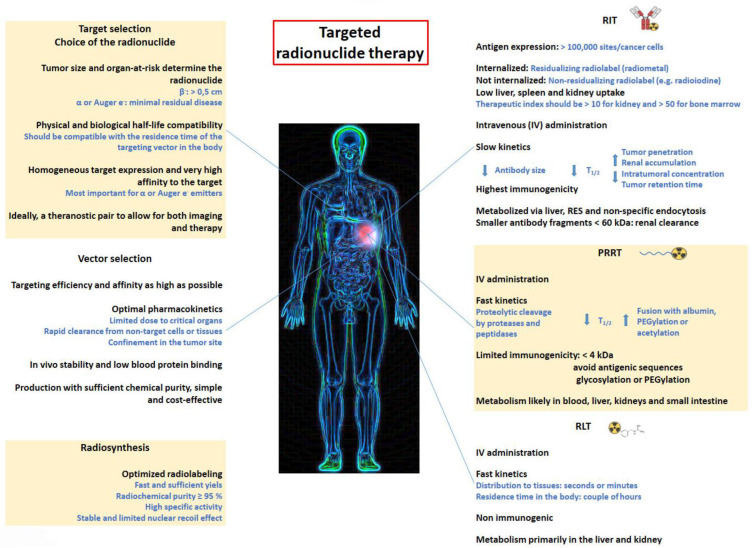
Overview of targeted radionuclide therapy considerations.

Until recently, RNT was generally considered only as a therapy of last resort and was not a part of the clinical referral process. In most cases, it has only been available in a few number of centers in the context of small clinical trials or for compassionate use [4,31]. The vast majority of investigated therapeutic radiotracers are in a preclinical stage. Yet, because of impressive outcomes against primary cancers as well as distant metastases, it is now accepted as a safe, effective and economically viable therapeutic modality. Targeted RNT is therefore receiving renewed attention from the oncology community as well as from the pharmaceutical firms [32]. An overview of the important considerations involved in developing targeted radionuclide therapies is given in Figure 3.

## 2. Radioimmunotherapy (RIT)

### 2.1. Antibodies and Derivatives

Although monoclonal antibodies have widely demonstrated their therapeutic potential in oncology and beyond, it is nevertheless possible to increase their effectiveness by coupling them to radionuclides [33]. The first historical use of radiolabeled antibodies in human was performed by William H. Beierwaltes in the 1950s which showed promising results [34]. Despite these encouraging results, it was not before the end of the 1970s that this new therapeutic modality started to rise, with Kohler and Milstein’s discovery of the hybridoma technology to produce monoclonal antibodies (mAb) specifically directed at tumor antigens [35]. The first clinical use of a radioimmunoconjugate was achieved for the localized irradiation of B-cell lymphoma [36]. The rationale of this therapeutic approach combining the specificity of mAbs associated with the cytotoxic effects of radionuclides is important and paved the way for RIT in cancer management.

Despite the success of this first RIT clinical trial, health regulators, concerned about potential dosimetric side-effects on healthy tissue, have slowed the development of this promising strategy. This initial indication (B-cell lymphoma) was the result of a judicious choice, considering both the high antigen overexpression and the radiosensibility of the tumor. In the 2000s, non-Hodgkin’s lymphoma (NHL) was the first major application of radiolabeled monoclonal antibodies. In this indication, two radioimmunoconjugates able to target the CD20 antigen have been approved by a number of national regulatory authorities: [^131^I]I-tositumomab (Bexxar^®^) and [^90^Y]Y-ibritumomab tiuxetan (Zevalin^®^). Clinical results obtained using Bexxar^®^ or Zevalin^®^ showed a significant efficacy and a long-term response [37]. Increase in knowledge about hematologic malignancies has allowed the development of new mAbs targeting other antigens, such anti-CD22 (epratuzumab), anti-CD37 (lilotomab) or anti-tenascin. These mAbs were radiolabeled with ^90^Y, ^131^I or ^177^Lu for clinical use in non-Hodgkin’s lymphoma trials [38,39,40,41].

**Figure 4:**
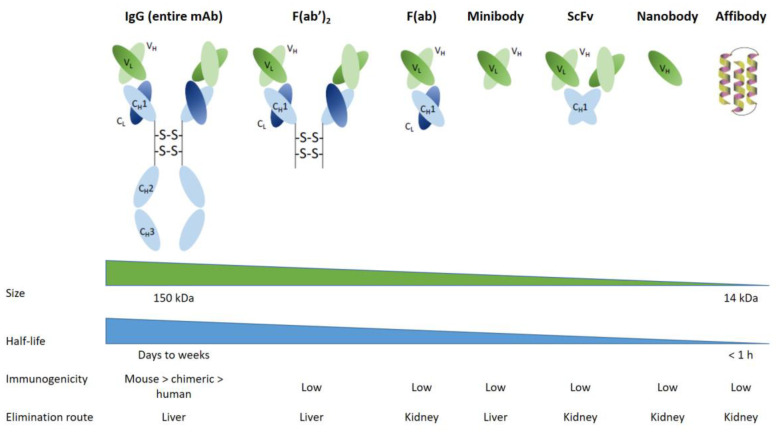
mAbs and derivates used in RIT procedures.

Subsequently, chimeric and humanized antibodies were developed as a result of biotechnological advances in mAb production. As was anticipated, these compounds have proven to be less immunogenic and have a better pharmacological tolerability profile for patients. The new properties of humanized mAbs allowed multiple injections and dose fractionation to increase overall tumor irradiation and improve the survival of healthy cells. However, mAbs have the pharmacokinetic disadvantages of being large molecules that diffuse relatively slowly and poorly within solid tumor, in addition with a slow clearance from blood and non-target tissues, hence, the use of long-lived radionuclides. In order to overcome these disadvantages, several approaches have been considered [42]. For instance, bioengineers and immunochemists have developed antibody fragments, such as F(ab) and F(ab’)_2_. Those smaller protein fragments, which conserve the specificity/affinity of entire mAbs, are produced by the reduction/digestion of initial mAb. In a recent clinical trial, an F(ab’)2 fragment targeting the sodium-dependent phosphate transport protein 2b (NaPi2b) was used in radioimmunotherapy for ovarian cancer. High efficacy in small solid tumors was demonstrated in this trial. Although F(ab’)2 is cleared by kidneys, side effects have been reported to be acceptable [43]. Based on this successful proof-of-concept for mAb fragments, bioengineering has led to the development of other small synthetic proteins (Figure 4), such as minibodies or fusion protein-like single-chain variable fragments (scFvs), which contain only a few parts of the entire mAb. Minibodies comprise CH3 (constant heavy 3) and VL/VH (variable light and variable heavy) moieties, whereas single-chain variable fragments (scFv) are composed of VL/VH moieties only. Minibodies and scFv are currently used for clinical imaging applications in nuclear medicine. Such engineered minibodies and scFv have been radiolabeled essentially with β^+^ radiotracers for PET imaging, but also with β- or α- radiotracers for radionuclide therapy [44]. Though therapeutic studies are still in the preclinical phase, studies on human has begun [45].

Single domain antibodies (also known as sdAb or nanobodies) have lately been developed from heavy chains antibodies found in camelids (dromedaries, camels, llama, etc.). They are composed of a monomeric part of the mAb VH fragment. It has now been possible to generate them from microbial hosts, and it appears that they will be used in future RIT clinical trials [46]. On the other hand, preclinical studies are underway on synthetic compounds consisting of three alpha-helix scaffold proteins (called affibodies) selected from a phage display library for their affinity to a specific antigenic structure [47].

### 2.2. Pretargeting Approach

The risk-to-benefit ratio of RIT is mainly driven by the ability to irradiate the tumor with a reduced dose to healthy tissues. To optimize the tumor cell delivery dose, pretargeting approach was developed in order to reconcile the relatively long pharmacokinetic half-lives of mAbs and the need for fast tumor biodistribution of the radionuclides. Pretargeting involves pre-injection of a non-radioactive bispecific antibody followed by injection a radiolabeled bivalent hapten peptide [48]. This protocol, known as “Affinity Enhancement System—AES”, allows to circumvent the slow clearance of the mAb. After determining the best time interval between the two injections (i.e., bispecific mAb and radiolabeled hapten), the hapten, designed to bind rapidly and specifically to the bispecific antibody pre-localized on the tumor, can be injected. The unbound haptens are rapidly excreted from the circulation via the kidneys, resulting in minimal exposure of healthy tissue.

In different clinical trials, promising therapeutic outcomes have been obtained using an anti-CEA pretargeted approach in metastatic medullary thyroid carcinoma [49,50]. The conclusion of these studies is a favorable balance between efficacy and toxicity, with a long-term stabilization of the disease.

### 2.3. Dose Fractionation Approach

One of the most frequent side effects of RIT concerns hematological toxicity. The recent arrival of humanized mAbs made it possible to consider the possibility of dose-fractionation with repeatable injections of the radiolabeled antibody. This dose-fractionation approach is well known in conventional external radiotherapy and allows a bone marrow regeneration between two injections. The dose-fractionation approach allows an increase in total injected dose compared to the classical single dose protocol, with an improvement in the clinical efficacy (in terms of progression free and overall survivals) without any increment in the toxicity [51,52,53]. Dose-fractionation should not be mistaken for retreatment, as it consists of delivering a higher cumulative dose of radiation without allowing for tumor repopulation between doses. Therefore, understanding and monitoring the treatment response represent a significant challenge, as all patients, for instance, will not require the same number of cycles [54]. Careful selection of the patients, predictive dosimetry and response assessment are crucial elements, and hence the growing role of theranostics, with the use of companion diagnostic agents [32,55].

## 3. Oligonucleotides

**Figure 5:**
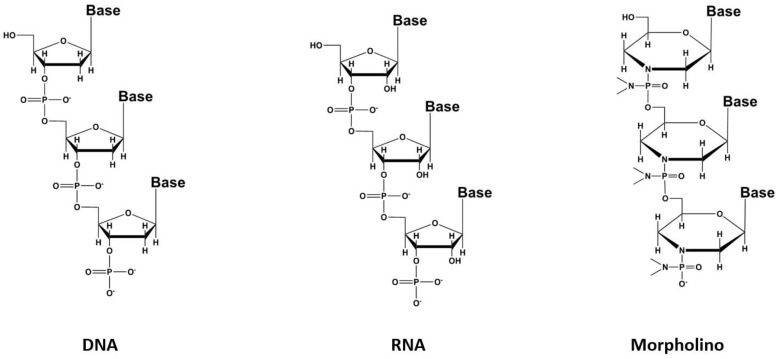
The different types of aptamers.

Oligonucleotides appear to be relevant in RNT, and are thus currently studied at the preclinical level. Oligonucleotides consist in short single-stranded oligomers of purine and pyrimidine bases. They exhibit the interesting capacity to bind to biological structures with high specificity, with the same order of magnitude than antibody–antigen binding (K_D_ is in nanomolar or sub-nanomolar range) [56]. These oligonucleotides are synthetic compounds and could be constituted by ribonucleotides (RNA aptamers) or deoxyribonucleotides (DNA aptamers). Aptamer compounds were developed in the beginning of the 1990s from a large library of randomly selected oligonucleotide sequences. The oligonucleotide sequence with the best affinity for a given biological target was selected in vitro using the SELEX method (Systematic Evolution of Ligands by Exponential Enrichment). The stability of these bioconjugates under several chemical conditions for radiolabeling, their ease of functionalization with various chemical moieties for the labeling with different radionuclides, coupled to their comparatively low production cost have been their major advantages over monoclonal antibodies. Initial investigations in nuclear medicine, and in preclinical studies, made use of aptamers for diagnostic applications, which confirmed these could be used as radionuclide vectors [57]. However, their use was rather limited due to the rapid renal clearance in vivo and the substantial instability towards nucleases. Several approaches have been developed to optimize these pharmacokinetic profile, such as the introduction of non-oligonucleotide patterns in the aptamer sequence, for instance, sugar rings, phosphodiester bonds or unnatural nucleotides in the single strand [58]. A recent promising improvement is based on the modification of the sugar chain with phosphorodiamidate morpholino oligomers (PMO) to produce compounds called morpholinos (Figure 5) with better pharmacodynamic and pharmacokinetic profiles [59]. The use of this class of conjugates for RNT has not yet reached the clinical stage but may represent an elegant solution, holding great promises in the near future, and their development thus deserves to be followed [60].

## 4. Peptide Receptor Radionuclide Therapy (PRRT)

Peptides, usually classified as containing less than 50 amino acids, are much smaller biomolecules. Peptides offer numerous advantages, including non-immunogenicity, favorable pharmacokinetics and simple production. On the other hand, their major limitations are possible nephrotoxicity due to high renal absorption and rapid in vivo degradation. Most natural peptides have a high affinity for their receptors, but, due to their rapid degradation, cannot be used to target for imaging or therapy. Peptides are nevertheless straightforwardly modified to improve stability, receptor affinity and permit the convenient grafting of various radiolabels [61,62,63]. However, the modifications of these small molecules to allow their labeling and improve their stability can strongly disturb their binding to the receptor if the amino acids essential for the binding have been modified or if the coupling of the chelating agent induces a phenomenon of steric hindrance. In the case of peptides, the difficulty therefore lies in obtaining molecules exhibiting a certain stability in vivo, while retaining sufficient affinity for cancer cells [64,65].

Many human tumors overexpress regulatory peptide receptors (Table 2), most of which belong to the G protein-coupled receptors (GPCRs) superfamily, a class of transmembrane receptors that are responsible for the transport of a variety of molecules across membranes. Peptides represent a particularly relevant class of molecules for medical use, particularly, but not exclusively, in nuclear oncology [66,67,68]. Many radiolabeled peptide derivatives are currently undergoing clinical trials around the world, one (a ^177^Lu-labeled somatostatin analog, Lutathera^®^) has even recently been approved for use.

### 4.1. The G Protein-Coupled Receptors Family

#### 4.1.1. Somatostatin Analogs

Somatostatin (SST) is a hormone peptide naturally present in the human body. It exists in two active forms of 14 and 28 amino acids, and is secreted by cells of the hypothalamus and also of the stomach, intestine and pancreas. Somatostatin has a regulatory function of the endocrine system, and plays a role in neurotransmission and cell proliferation. To play its role, somatostatin binds to five membrane receptors subtypes named SSTR1 to SSTR5. Those receptors are widely expressed in healthy tissues, with diverse levels. In several tumor contexts such as gastroentero-pancreatic neuroendocrine tumors (GEP-NET), as well as pituitary adenomas and some other malignancies (e.g., hepatocellular carcinomas, lymphomas, small cell lung cancers, etc.), SSTRs are overexpressed [69]. Therefore, many somatostatin analogues have been developed to target these membrane receptors for therapeutic purposes, with mixed results, except for neuroendocrine tumors, for which those analogs have been approved (Table 3). Many somatostatin analogs have already been labeled with various radioelements, whether for imaging or for therapy, with probes used today in routine clinical applications, and more compounds that are in the clinical stage [70]. Four somatostatin analogs are currently approved for neuroendocrine tumor imaging, [^111^In]In-Pentetreotide ([^111^In-DTPA^0^]-octreotide, Octreoscan^®^), now increasingly replaced by [^68^Ga]Ga-DOTATATE (NETSPOT^™^), [^64^Cu]Cu-DOTATATE (Detectnet^™^), both approved in the US, and [^68^Ga]Ga-DOTATOC (Somakit TOC^®^) in both Europe and the US [71]. On the therapeutic side, [^177^Lu]Lu-DOTATATE (Lutathera^®^) has been approved in well-differentiated, unresectable or metastatic, progressive midgut neuroendocrine tumors, both in Europe and in the US [72].

PRRT with somatostatin analogs has been widely reviewed [70,73,74,75,76]. Eventually, two beta emitters derivatives, [^90^Y]Y-DOTATOC (Octreother^®^) and [^177^Lu]Lu-DOTATATE (Lutathera^®^), have demonstrated their clinical utility. Yttrium-90 is a high-energy β^−^-emitter without gamma emission, and consequently present the associated disadvantage of a large tissue penetration that lead to healthy tissue irradiation without the capacity of resolutive image for the follow-up and dosimetry of the peptide behavior. To circumvent these disadvantages, lutetium-177 has gradually taken a major place in PRRT approach over recent years. Indeed, the latter presents a shorter tissue penetration (2.2 mm compared to 12 mm for yttrium-90; cf. Table 1) and associated gamma emissions (208 keV at 10.4% and 113 keV at 6.2%) that allow a biodistribution monitoring of the peptide. The successful NETTER-1 Phase 3 trial with [^177^Lu]Lu-DOTATATE can be considered as the cornerstone of PRRT, which established it as a treatment modality to be considered, opening the way for a truly vibrant research area [77]. Current and extensive research with [^177^Lu]Lu-DOTATATE aims to improve the safety and efficacy of this PRRT and allows the identification of prognostic and predictive factors to optimize patient care management, and thereby to enlarge possible indications [78]. In parallel, α-labeled somatostatin analogs (either DOTATOC or DOTATATE) were shown to outperform ^177^Lu [79,80,81].

Unexpectedly, but with increasing evidence, antagonist analogs have been proven to outperform agonist ones (higher tumor uptake, better dosimetry profile, and faster clearance), despite the absence of internalization of the ligand–receptor complex [82]. This higher tumor uptake is a direct consequence of the presence of more target binding sites for antagonists associated with a more slow dissociation than for agonists. This may notably widen applications to targeting to tumors with lower receptors expression [83]. Preclinical and clinical studies have confirmed the potential superiority of antagonist-based tracers [84]. However, the firstly SSTR antagonist developed ([^111^In]In-DOTA-BASS) has shown only a very modest affinity for the principal expressed SSTR subtype (SSTR2) in neuroendocrine tumor. To go around this issue, a second generation of this class of biomolecules was designed (LM3, JR10, and JR11), with improved pharmacological characteristics (cf. Table 4) [85]. A pilot study first compared [^177^Lu]Lu-DOTA-JR11 ([^177^Lu]Lu-satoreotide tetraxetan) with [^177^Lu]Lu-DOTATATE. The results showed a higher tumor dose up to 10 times greater for the antagonist compound [86]. As a consequence, a phase I study was performed. This clinical trial included 20 refractory and well-differentiated NET patients who have been treated with an activity of 7.4 GBq per 3 months, six patients receiving one cycle and fourteen receiving two cycles. Preliminary results were encouraging, with a response rate of 45%, a disease control in 85% of patients and a median progression-free survival of 21 months. There were, however, some safety concerns, since grade 4 myelosuppression was observed in 57.1% (4/7) of cases after the second cycle, requiring a protocol amendment to reduce the activity of the second cycle by 50%, in order to limit the bone marrow dose to 1 Gy. [87]. [^177^Lu]Lu-DOTA-LM3 has been studied in 51 NET patients using the same dosimetry protocol as for [^177^Lu]Lu-DOTATATE. Once again, antagonist compound showed a higher tumor dose and disease control was observed in 85% of patients, without severe adverse effects, except for thrombocytopenia in a few patients [88]. Current developments thus logically focus on the use of α-emitting nuclides and a switch from agonist to antagonist somatostatin analogs [89,90].

#### 4.1.2. Bombesin Analogs

Gastrin-releasing peptide receptors (GRPr) are a subtype of the bombesin receptor family, overexpressed in several malignancies, such as lung, prostate, breast, gastric and colorectal carcinomas [91]. Their activation stimulates both cell growth and proliferation. Bombesin is a natural 14-amino acid peptide isolated from the frog *Bombina bombina* with a strong-affinity for GRPr. Its 27-amino acid mammalian version, named GRP, shares the same final seven amino acids sequence (Trp-Ala-Val-Gly-His-Leu-Met-NH_2_), used in the receptor recognition. Several radiolabeled bombesin derivatives have thus been developed and investigated as a useful tool for the detection and/or treatment of cancers [92]. To date, bombesin derivatives have been mainly investigated in clinical studies for imaging approaches. Recently, bombesin derivatives have been the subject of therapeutic clinical studies after radiolabeling with ^177^Lu, but several preclinical studies with other β^−^ and α-emitters have been reported [93].

The first, and still the most widely tested, therapeutic analog is [^177^Lu]Lu-AMBA ([^177^Lu]Lu-DOTA-Gly-4-aminobenzyl-BBN(7–14)). [^177^Lu]Lu-AMBA has initially been investigated in a phase I study, with seven patients who suffered from hormone-refractory metastatic prostate cancer, obtaining disappointing results, because of a low plasma stability and strong pancreas uptake [94]. As with somatostatin analogs, bombesin antagonists have been reported to outclass agonists [95,96]. GRPr antagonists, RM2 and NeoBOMB1 (now rechristened NeoB) (Table 4), were labeled with ^68^Ga for imaging breast and prostate cancer patients. Results displayed high contrast for the tumor lesions, despite lower internalization. Therapeutic applications with 4.5 ± 0.9 GBq of [^177^Lu]Lu-RM2 have been considered in a first-in-human dosimetry study [97]. Some preliminary results in four patients suffering from mCRPC showed a mean absorbed dose of 6.20 ± 3.00 Gy/GBq in the tumor lesions without side effects. Major risk organ appears to be the pancreas. A derivative with improved stability and affinity, [^177^Lu]Lu-AMTG ([^177^Lu]Lu-α-Me-l-Trp^8^-RM2), has recently been reported [98]. [^177^Lu]Lu-NeoB is currently investigated in a phase I/II study in patients with advanced solid tumors (NCT03872778). The patients were selected after preclinical evaluation for GRPr expression [99,100]. The preliminary results with these antagonists showed a need for further optimization, particularly regarding safety [93]. Lately, an original ^67^Cu-labeled antagonist derivative has been reported, [^67^Cu]Cu-SAR-BBN, demonstrating encouraging tumor inhibition in mice bearing prostate carcinoma model [101]. Of note, a clinical trial is currently underway in Australia with the homologous diagnostic, [^64^Cu]Cu-SAR-BBN, in metastatic ER+/PR+/HER2− breast cancer (ACTRN12619001383156).

#### 4.1.3. Substance P

Neurokinin (or tachykinin) receptors are located in the central and peripheral nervous system and possess three subtypes, NK_1_, NK_2_ and NK_3_. Among them, NK_1_ has been proven to be overexpressed in primary malignant gliomas [102]. Physiologically, substance P (SP), an 11-amino acid neuropeptide, is an endogenous ligand for NK_1_ receptor. As a consequence, this neuropeptide appears as an interesting targeting agent for brain tumors imaging and therapy [103].

Initial results in some pilot studies with ^90^Y or ^177^Lu-labeled SP demonstrated potential clinical therapeutic indications. The growing interest towards α-particle-emitting radionuclides, particularly the short-lived ^213^Bi, shows encouraging preliminary results in terms of feasibility, efficacy and safety [104]. The use of 1.4 to 9.7 GBq of [^213^Bi]Bi-DOTA-SP has demonstrated a median 5.8 months progression free-survival and 16.4 months overall survival time [105]. The radiotracer has been administered via a surgically implanted catheter inside the tumor or, after its resection, in the resultant cavity, to enable to pass the blood–brain barrier and improve tumor uptake. Currently, preclinical and early clinical studies are ongoing with other alpha-emitters, such as ^211^At- or ^225^Ac. These innovative alpha-emitters radionuclides present the advantage of longer half-lives than ^213^Bi, respectively, 9.9 days for ^225^Ac, 7.2 h for ^211^At and 46 min for ^213^Bi.

Preliminary results of a twenty patients dose-escalation study with [^225^Ac]Ac-DOTAGA-SP appears very promising for the therapy of high mortality glioblastoma [106]. Current research seems to focus on NK_1_R antagonists [107,108].

#### 4.1.4. Other Analogs

G protein-coupled receptors form a large superfamily of cell-surface receptor. They represent a common large group of protein expressed in eukaryotes cells. Some of these receptors appears to be a credible alternative—or supplementary—target for nuclear medicine purposes [109]. Several promising results from preclinical and early clinical studies involving the utilization of radiolabeled peptides, targeting receptors, such as glucagon-like peptide-1 (GLP-1), melanocortin type 1, neuropeptide Y, neurotensin, or vasoactive intestinal peptide, have been documented. [110,111,112]. Cholecystokinin-2 receptor is, for instance, of particular interest, especially in medullary thyroid carcinoma, and can be specifically targeted with gastrin-derived peptides [113]. Initial findings were reported regarding the use of [^177^Lu]Lu-PP-F11N ([^177^Lu]Lu-DOTA-(D-Glu)_6_-Ala-Tyr-Gly-Trp-Nle-Asp-PheNH_2_), a mini-gastrin analog labeled with ^177^Lu, for treating advanced medullary thyroid carcinoma in patients. According to the dosimetry results, it has been found to be a viable tool for therapy. However, stomach was the organ-at-risk, while absorbed doses in the kidney and bone marrow were low. [114]. There is therefore a wide field of possible investigations in this area of research.

### 4.2. C-X-C Chemokine Receptor Type 4 (CXCR-4)

**Figure 6:**
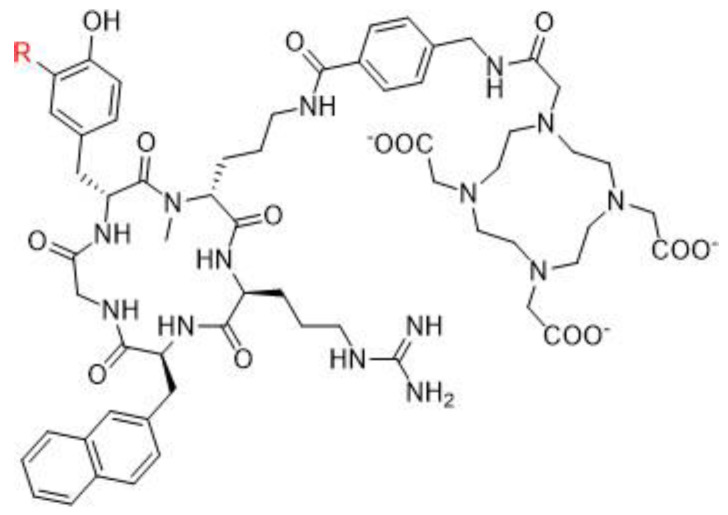
CXCR4-targeting peptide derivatives. R = H: Pentixafor^®^, R = I: Pentixather^®^.

Chemokines are involved in many physiological and pathological processes linked to cell homing and migration. In this large chemokine family, the CXCR4 protein (also called fusin or CD184) is widely studied in nuclear medicine for RNT of cancers. CXCR4 is an alpha chemokine receptor for the stromal-derived factor-1 (SDF-1, also called CXCL12) initially discovered for its involvement in HIV infection. CXCR4 is naturally present in the blood cell lineage from hematopoietic stem cells (HSCs) and hematopoietic progenitor cells (HPCs), while stromal cells secrete CXCL12. The interplay between CXCL12 and CXCR4 plays a crucial role in regulating several hematological processes, such as homing, quiescence, and retention of HSCs and HPCs in the bone marrow microenvironment [115]. More recently, studies have shown that the CXCR4 protein is expressed in over 20 types of human hematological and solid cancers. [116,117]. Tumor growth, metastasis, angiogenesis, relapse and therapeutic resistance have been correlated with an overexpression of CXCR4. Accordingly, CXCR4 could be a target of great interest for imaging (diagnosis and following) as well as in a therapeutic perspective [118]. Over the past two decades, several CXCR4 binding peptides have been developed, such as the T140 series (linear 14 amino acid CXCR4 antagonist peptides), AMD3100 derivatives (bicyclam organic compounds acting as an inhibitor of CXCR4 function), and FC131 compounds (cyclopentapeptides also presenting a CXCR4 antagonistic activity). More recently, attention has shifted towards the development of another peptide, FC231, a small cyclic pentapeptide derivative presenting great promises for targeting CXCR4 for radiotherapeutic applications [119]. At the preclinical stage of FC231 derivatives, correlation between CXCR4 affinity and modifications in chemical structure were found. Through an extensive screening, the optimal peptide/chelator/radiometal combination in order to proceed to a radiopharmaceutical was established. To preserve the best affinity despite the radiolabeling, the chemical scaffold was adapted for imaging ([^68^Ga]Ga-Boclatixafortide; Pentixafor^®^) or therapy ([^177^Lu]Lu-Anditixafortide; Pentixather^®^) [120] (Figure 6).

Radiolabeled with β- emitting nuclides, as ^90^Y or ^177^Lu, Pentixather^®^ has shown very promising results for poor prognosis patients with advanced multiple myeloma [121]. This success, coupled with the ubiquitous CXCR4/CXCL12 axis in the development of various hematologic and solid tumors, indicates a potential widespread use of CXCR4 targeting in RNT. Ongoing clinical investigations are being carried out to determine the safety and efficacy profile of CXCR4-targeted RNT. Typical side effects observed so far were blood dysplasia and kidney failure due to tumor lysis syndrome, while hepato- and overall nephrotoxicities remained limited [122]. Despite these side effects, CXCR4 has proven to be safe and well-tolerated for therapeutic approaches in various clinical situations [123,124,125].

### 4.3. Other Peptide Derivatives

Several other peptide-based targeting agents have shown promising results for therapeutic purposes, such as integrins, especially the cell adhesion receptors αVβ_3_ and αVβ_5_. These integrins are notably engaged in various tumor processes, such as angiogenesis, proliferation, survival and metastasis [126]. The triple amino-acids sequence Arg-Gly-Asp (RGD), being specifically recognized by αVβ_3_ receptors, has led to the development of numerous radiolabeled RGD peptides, including dimeric compounds, with improved affinity [127]. These have been labeled with ^90^Y, ^177^Lu, and ^188^Re for therapeutic purposes, but none of these compounds have been used in clinic yet. A recent paper by Notni aims at explaining the reasons for the lack of clinical success for radiolabeled RGD peptides [128]. It has been postulated that other integrins might be more suitable for tumor targeting [129]. Another receptor, vascular endothelial growth factor receptor (VEGFR), which is particularly involved in the tumor neo-angiogenesis, could be targeted with radiolabeled peptides to affect the angiogenesis process [27]. Another membrane receptor related to vascular endothelial growth factor receptor which may be targeted with radiolabeled peptide is neuropilin-1, expressed by several tumors, for instance, glioma. A ^177^Lu-labeled peptide targeting NRP-1 has recently been reported, but need some more optimization before being considered further [130].

The expertise field concerning radiolabeling peptidic analog development is highly active, with an important increase in the number of reports [131,132]. A new method being explored is the development of peptides that target highly specific proteins in tumors, such as glypican 3 for hepatocellular carcinoma. [133]. Another strategy is to target different tumor compartments, such as ECM and TAMs. Innovative tools, such as cell-penetrating and tumor-homing peptides, also clearly deserve attention as putative radiopharmaceutical candidates. All of these potential avenues are currently being studied. [134].

## 5. Radioligand Therapy (RLT)

Compared to antibody- or peptide-based radiopharmaceuticals, small molecules present several pharmacological and pharmacokinetic benefits. They are typically less expensive to produce and have faster pharmacokinetics due to their lower molecular weights and higher lipophilicity. Furthermore, they can be radiolabeled in more stringent conditions (higher temperatures or broader pH range). Recent developments in non-peptidic antagonists have shown promising biological and physicochemical properties. These compounds, such as [^177^Lu]Lu-3BP-227, a potent neurotensin receptor 1 antagonist, have shown encouraging clinical results in a proof-of-concept clinical trial in pancreas cancer (Table 4) [135]. In a similar manner, though it has yet only been demonstrated in vitro or in mice models, ^177^Lu-labeled non-peptidic NK_1_R and CCK_2_R antagonists exhibited better binding characteristics than their respective agonists counterparts [108,136]. In addition, many indications take advantage of radioligand therapy with small organic molecules or metallic radiocomplexes.

### 5.1. Bone-Seeking Agents

Bone metastases are a very common complication in several cancer types. They notably occur in approximately half the cases of breast carcinoma, the primary cancer in females, and in 80% of men with prostate adenocarcinoma, the second most common one in males [137]. The term skeletal metastasis covers two entities: on the one hand, the infiltration of the bone marrow, which represents probably the first structure invaded, and, on the other hand, the involvement of the bone matrix, most often secondary to the first. Tumor infiltration is directly responsible for the pain phenomenon. Targeted radionuclide therapy can offer a significant therapeutic alternative to relieve pain for the patients [138]. Increased osteoblastic activity under the effect of metastatic tumor cells is responsible for the hyperfixation of the radiotracer. Consequently, all localizations are treated immediately by means of a single intravenous injection, even in the case of plurifocal lesions. However, there is no specificity of the fixation, which is solely correlated with vasculature; any hypervascularization of the tissues induces an hyperfixation of the radiotracer into those tissues. Initial use of therapeutic radionuclides to treat the pain of bone metastases relied on [^32^P]-orthophosphate or [^32^P]-biphosphonate, which are molecules having a very strong affinity toward calcium present in the actively growing bone [139,140]. Unfortunately, ^32^P has a high hematological toxicity because of its significant dose delivered to bone marrow.

Subsequently, a large variety of radiopharmaceuticals able to deliver radiation to metastatic bone sites have been developed [140,141,142,143,144,145,146,147,148] (Table 5). Among all those developed compounds, four are currently commercially available: [^89^Sr]SrCl_2_ (Metastron^®^) and [^223^Ra]RaCl_2_ (Xofigo^®^), taking advantage of the Sr^2+^ and Ra^2+^ ions’ mimicking the Ca^2+^ cation, and thus having a natural tropism for the inorganic bone matrix, and [^153^Sm]Sm-EDTMP (Quadramet^®^), and [^186^Re]Re-HEDP ([^186^Re]Re-etidronate^®^), where the radionuclides are used as phosphonates (EDTMP = ethylenediaminetetramethylene phosphonate and HEDP = hydroxyethylidene diphosphonate), binding to the hydroxyapatite of bone surface, then penetrating deeper through remodeling of the matrix. To date, [^223^Ra]RaCl_2_ is the only one with a proven benefit on overall survival [146]. However, [^188^Re]Re-HEDP, replacing rhenium-186 with its rhenium-188 congener, might prove itself as clinically useful. Indeed, there has been some evidence that the latter could also lead to an improvement in overall survival. In a retrospective analysis, based on a former phase 2 trial, repeated injections (from 1 to 3) were correlated with a gain in survival from 4.50 to 15.66 months [149,150]. A phase 3 trial, aiming at comparing the respective efficacies of [^188^Re]Re-HEDP with [^223^Ra]RaCl_2_, in patients with castration-resistant prostate cancer metastatic to bone is currently ongoing (RaRe trial, NCT03458559).

Beside rhenium-188, lutetium-177 is another potentially interesting radionuclide for bone-pain palliation [151]. Several clinical studies investigating [^177^Lu]Lu-EDTMP and [^177^Lu]Lu-DOTA^ZOL^ (^177^Lu-zoledronic acid) have been published, warranting further research [152,153]. Nevertheless, with the advent of the radiotherapeutic PSMA derivatives, the question of the future clinical utility of these bone-seeking agents for the management of metastatic prostate cancers arises, though its interest remains for bone metastases arising from other tumors, such as breast or lung carcinomas.

### 5.2. [^131^I]mIBG

Firstly, designed to allow adrenal medulla imaging, meta-iodobenzylguanidine (mIBG) or iobenguane, is an aralkylguanidine radioiodinated in a meta-position of its benzene moiety [154]. This physiological analogue of the noradrenaline (or norepinephrine) neurotransmitter is not metabolized and could be excreted without any modification unlike adrenaline. It could also be gathered by cell through either non-specific and non-saturable passive diffusion in all cells, or an active, specific, and saturable uptake in cells expressing the norepinephrine transporter, with high-affinity. This active absorption is reported in many tumors, especially those of neural crest and neuroendocrine origin (neuroblastoma, carcinoid tumors, paraganglioma, and pheochromocytoma) and is about 30 times more efficient than passive transport [155].

Commercially, mIBG is available, either labeled with iodine-123 (^123^I) for the purpose of imaging, or with iodine-131 (^131^I) for the purpose of therapy or, rarely, for imaging as well. [^131^I]mIBG is synthesized by isotopic exchange with radioiodine, resulting in a consequent amount of unlabeled mIBG (low specific activity, LSA) in the radiopharmaceutical product. This cold product can enter in competition with radiolabeled [^131^I]mIBG, restraining tumor cell uptake. However, another kind of process enables to yield no-carrier-added (n.c.a.) [^131^I]mIBG, with a very high specific activity (1600 mCi/mol, HSA) allowing a more potent therapeutic dose that is less likely to saturate receptors on tumor cells, thereby resulting in an increased cytotoxic activity [156]. It has been reported, that repeated low-specific activity [^131^I]mIBG injections might be effective in controlling tumor expansion [157]. In relapsed and refractory neuroblastomas, a median remission rate of 30% has been reported [158]. Different dosing regimens have been proposed. EANM has established procedure guidelines to select best responding patients and harmonize protocols [159]. [^131^I]mIBG treatment is generally well tolerated, although high-dose treatment regimens (18 mCi/kg) are susceptible to conduce to a myelotoxicity. In this case, it is followed by autologous stem cell transplantation [160]. The maximum tolerated dose without hematological toxicity was found to be 12 mCi/kg. It has been used in combination with high-dose chemotherapy and total-body EBRT, to eliminate residual disease or to shrink the primary tumor enabling surgical resection [161].

There have been clinical studies demonstrating the effectiveness of both HSA and LSA [^131^I]mIBG as a therapeutic agent since the 1980s, and it has received approval in both the US and Europe [162]. However, there have been only limited randomized controlled trials and its availability has often been problematic, which has hindered its clinical use for the benefit of [^177^Lu]Lu-DOTATATE, that has replaced it in some indications [157,163]. Indeed, HSA [^131^I]mIBG (Azedra^®^) was FDA approved in metastatic paragangliomas, and granted orphan drug designation, on the basis of only one single successful clinical trial, single-arm and open-label, in 68 patients [164]. Nevertheless, [^131^I]mIBG is still under clinical investigations. Recently, the potential use of Auger-emitting ^125^I or α-emitting ^211^At to replace ^131^I for the treatment of residual or metastatic pheochromocytomas and paragangliomas has been explored [165,166] and a first-in-human trial of [^211^At]mABG was recently introduced [167].

### 5.3. Prostate-Specific Membrane Antigen (PSMA) Inhibitors

**Figure 7:**
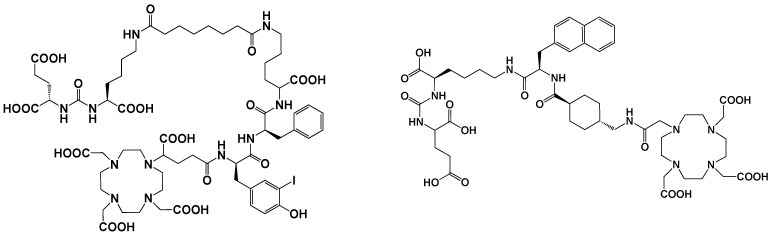
PSMA-I&T (**left**) and PSMA-617 (**right**) structures.

Originally discovered in the 1990s, PSMA is an antigenic transmembrane glycoprotein presenting two enzymatic activities, acting as glutamate carboxypeptidase and folate hydrolase. Firstly, it has been described as a prostate specific enzyme, located in the cytosol of the physiologic prostate cells. In prostate cancer context, PSMA becomes an overexpressed transmembrane bound protein [168]. PSMA is not a secreted protein and it appears to have an upregulation in patients treated by an androgen deprivation therapy; metastatic lesions of prostate cancer also appear to be PSMA positive. In recent works, PSMA have been identified to be expressed in other malignancies as well, particularly in the neovasculature of tumors, such as glioblastoma, renal cell carcinoma, hepatocellular carcinoma, lung cancer or breast cancer [169,170,171]. Initially, biologists created monoclonal antibodies, such as 7E11-C5.3 and HuJ591, directed towards PSMA. Currently, the clinical use of PSMA in nuclear medicine is based on small molecules that target PSMA enzyme activity, with better pharmacokinetic profile than antibodies, i.e., faster tumor uptake coupled with faster kidney elimination. Consequently, they present better dosimetric and efficacy profiles [172]. The small molecules which target PSMA are urea-based enzyme inhibitors, and can be used for diagnosis and radionuclide therapy. The current development of PSMA inhibitors allows a theragnostic approach with PET imaging and therapeutic using, respectively, gallium-68 and lutetium-177. To achieve this radiolabeling, DOTAGA-chelator moiety was added to PSMA molecules in order to functionalize it; this new compound is dubbed PSMA-I&T (for imaging and therapy). To improve the pharmacokinetic profile (decreasing kidney and salivary gland absorption), some modifications were performed to the PSMA-targeting molecules, leading to the creation of PSMA-617 (Figure 7). While preclinical studies in mice demonstrated that PSMA-617 had better renal dosimetry than PSMA-I&T, this advantage was not observed in humans and the two molecules appear to be quite similar [173]. Despite this renal uptake, the phase III global VISION clinical trial (NCT 03511664) showed promising results for [^177^Lu]Lu-PSMA-617 in RNT of metastatic castration-resistant prostate cancer (mCRPC) [174]. In terms of toxicity, the most exposed organs are kidneys, but they could be protected effectively by co-injecting an amino-acid solution, followed by salivary glands leading to dry mouth syndrome, lacrimal glands and bone marrow. All of these adverse effects are transient and do not present a severe degree. The principal serious side effect is hematological symptoms of grade ≥ 3 which was reported in 12% of treated patients [175]. Phase III global VISION clinical trial displayed major prolongations of imaging-based progression free and overall survivals, respectively, 5.3 months (median 8.7 vs. 3.4 months) and 4.0 months (median 15.3 vs. 11.3 months).

Consequently, PSMA-targeting is rising in RNT and appears to be promising in the care management of metastatic castration-resistant prostate cancer. Nevertheless, approximatively 30% of patients do not respond to the [^177^Lu]Lu-PSMA-617 RNT. α-particle emitting radionuclides, such as ^225^Ac or ^213^Bi, have been used to radiolabel PSMA-617, in order to increase the anti-tumor activity, and to treat relapsed patients with no major risk of side effects. First clinical use of [^225^Ac]Ac-PSMA-617 or [^213^Bi]Bi-PSMA-617 displayed images of complete response with no major hematologic toxicity. Only observed side effect was xerostomia due to the salivary gland uptake [176,177]. These preliminary results of α-RNT are promising and could be an alternative for advanced staged patients escaping [^177^Lu]Lu-PSMA-617. However, a large clinical trial have to be conducted to confirm these results.

### 5.4. Fibroblast-Activation Protein (FAP) Inhibitors

Cancer-associated fibroblasts (CAFs) are the most abundant cell type inside the tumor microenvironment. They are characterized by the expression of fibroblast activation protein-α (FAP), a type II membrane-bound glycoprotein serine protease [178]. The latter is highly expressed on the cell surface of activated fibroblasts but not on quiescent ones. Of note, more than 90% of epithelial cancers’ stroma are expressing FAP. In addition, FAP has notably been linked with a poor prognosis by enhancing tumor growth, matrix remodeling and angiogenesis. More than 28 tumor types have been proven to strongly fix a FAP-targeted ^68^Ga-radiopharmaceutical [179]. Consequently, this new target has driven a high interest for therapy as well as for imaging [180].

**Figure 8:**
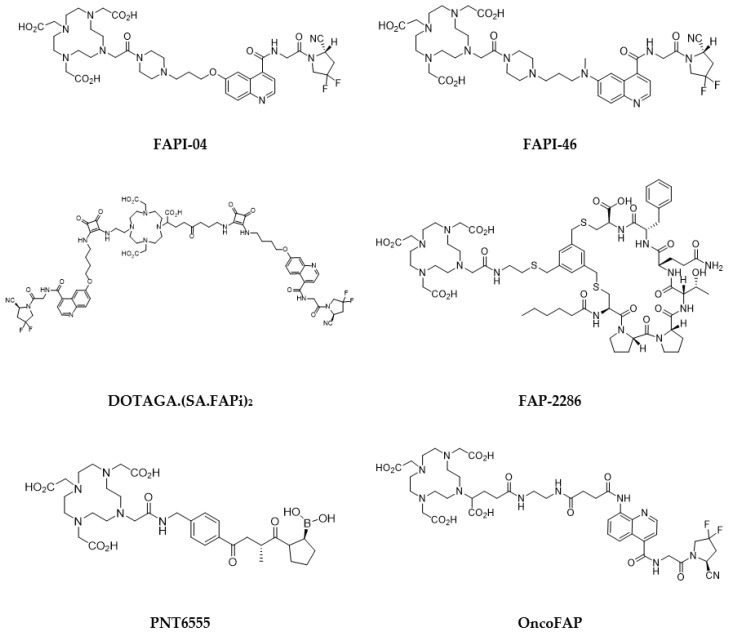
FAP inhibitors of interest for targeted radionuclide therapy.

Quinoline-based small molecules that function as FAP inhibitors (FAPis) have demonstrated highly promising outcomes in both preclinical and clinical investigations [181,182]. These molecules may also be the first pan-cancer theranostic agent. Multiple clinical studies have been conducted using [^68^Ga]Ga-FAPI-04, and it exhibited outstanding uptake in tumors, displaying high sensitivity and image quality across various tumors, though the evidence of some potential false-positive results may raise concerns [179,183]. Use of the ubiquitous DOTA chelator allows to substitute the diagnostic-aimed ^68^Ga with therapeutic nuclides, i.e., ^90^Y or ^177^Lu. Radiolabeled FAPi derivatives have thus been investigated in proof-of-concept studies for potential therapeutic application, though, to date, no large clinical trial has been initiated. FAPI-04 appears to be limited by rapid clearance from tumor, resulting in a limited radiation dose [181,184]. Further derivatives were developed, such as FAPI-46 (Figure 8). Initial investigations in small cohorts with ^90^Y- and ^177^Lu-labeled FAPI-46 displayed encouraging results, since treatment was well tolerated, while it some clinical response was also observed [185,186,187,188,189,190]. On the contrary, labeling with the β^—^ emitter of medium-energy samarium-153, led to poor results due to low-specific activity in one case report with sarcoma [191]. Yet, further clinical application may be challenging because of a too fast clearance and an insufficient tumor retention.

To improve pharmacokinetic profile, and subsequently therapeutic effectiveness of these radiotracers, some ligand optimization has been performed. One strategy has been to attach an albumin-binder to extend circulation and increase tumor accumulation, with apparently encouraging results [192,193,194]. Another proposed strategy has been the use of dimeric agents [195,196]. A first-in-human study with a ^177^Lu-labeled squaric acid-derivatized dimeric FAPi derivative showed it was well tolerated and displayed a high tumor uptake [197]. This was further confirmed in a subsequent pilot study in 15 patients with iodine-refractory differentiated thyroid carcinoma [198].

The evaluation of novel labeled FAP-targeting agents in first-in-human studies has been initiated with compounds, such as FAP-2286, a DOTA-modified FAP-binding peptide chelator, and PNT6555, also a DOTA-based radiotracer, with a boronated FAP-targeting moiety, all radiolabeled with ^177^Lu (Figure 8). Only results regarding FAP2286 have been currently reported [199]. Eleven patients with various primary tumors were treated with 5.8 ± 2.0 GBq of [^177^Lu]Lu-FAP-2286. It demonstrated long retention in the tumors, with acceptable side effects. Another FAP ligand, with ultra-high affinity, in the subnanomolar range (680 pM, vs. 6.5 nM for FAPI-04), has newly been reported, and labeled with ^177^Lu [200]. This radiotracer (OncoFAP) demonstrated a favorable in vivo biodistribution in tumor-bearing mice [201]. Its dimer derivative was demonstrated to display an even more stable and prolonged tumor uptake, with 20% ID/g vs. 4% ID/g 24 h post-injection, respectively [202]. More studies need to be conducted to confirm their clinical usefulness.

Stromal and malignant cells in the primary tumor could be targeted using the cross-fire effect, radiolabeling FAPi with β^−^-emitting radionuclides. Otherwise, α-particle or Auger electron emitting radionuclides’ short range could be exploited to target pro-tumorigenic CAFs while avoiding tumor-suppressive CAFs. For this purpose, [^225^Ac]Ac-FAPI was developed and evaluated in a pancreatic carcinoma model, displaying a rapidly emerging therapeutic effect but lasting less than its ^177^Lu-labeled counterpart [203]. However, the therapeutic effect was limited, leading to the suggestion that FAPI labeled with ^188^Re or ^211^At might be more appropriate. Yet, no ^211^At-labeled FAPi compound has been described so far. FAPI-34, on the other hand, has been reported as a FAPi ligand amenable for radiolabeling with ^188^Re, though its radiolabeling has only been reported for the homologous ^99m^Tc right now [204].

There have been relatively few clinical studies conducted on FAP-targeted radionuclide therapy, and the results have been varied, which may be due to the multiple tumor types and patient conditions evaluated. Further studies on ligand design is necessary. Additionally, research is required to establish the efficacy of this therapy, and it has been recommended that its use in combination with other treatment modalities, for instance, immunotherapy, should be explored as it may provide a better efficacy [205,206].

### 5.5. Poly(ADP-Ribose)Polymerase (PARP) Inhibitors

Poly(ADP-Ribose)polymerase (PARP), which are vital DNA repair enzymes associated with chromatin, are prominently expressed in the nucleus of mammalian cells. Due to the high rate of proliferation and replicative stress, tumor cells are susceptible to genomic instability, resulting in the overexpression of PARP, unlike normal ones [207]. Targeting of PARP enzymes has therefore recently emerged as potentially attractive to selectively target tumor tissues [208]. Blocking the expression of PARP, particularly PARP1, disrupts replication and leads to cell death, with a greater effect on faster-growing malignant cells. Additionally, PARP inhibition has been used as a chemo- or radiosensitizer in combination therapies [209]. In recent years, various PARP1-targeted inhibitors (PARPIs), such as olaparib, talazoparib, and rucaparib, have been approved for use. They have shown promises in preventing DNA repair after exposure to external-beam radiotherapy or radioimmunotherapy. To optimize the benefits of this treatment modality, it is crucial to non-invasively determine the level of PARP1 expression, as it could help predict the response to PARP inhibitor therapies [210]. Several radiolabeled PARPIs have thus logically been reported, mostly olaparib or rucaparib derivatives, both for imaging and therapy [211]. Alpha particles and Auger electron emitters are considered more appropriate, although there have been some descriptions of radiotracers labeled with beta particle emitters [212]. Latest efforts in the domain have been recently reviewed [213].

PARPis are a type of small organic molecules that are often preferred to be labeled with radiohalogens as they do not require any chelator in contrast to radiometals [214,215]. In a preclinical study, using a neuroblastoma model, [^211^At]At-MM4, a rucaparib derivative labeled with an alpha emitter, demonstrated favorable uptake and a significant increase in median survival, especially with multiple fractionated doses, while toxicity remained limited [216]. On the other hand, an analog of [^211^At]At-MM4 labeled with an Auger emitter, [^125^I]I-KX1, was found to be less effective but could potentially be useful in treating micrometastatic neuroblastoma [217]. Another Auger-emitting PARPi, [^123^I]I-MAPI, also demonstrated an increase in survival with limited toxicity in mice with glioblastoma multiforme, a type of brain cancer with a very poor prognosis [218]. Its main limitation is its inability to cross the blood–brain barrier, that could be overcome with the use of nanoparticles. These findings suggest that PARPis labeled with alpha or Auger emitters have potential as a targeted therapy for various cancers. Pharmacokinetic profiles seem to be the major limitation of these compounds, since [^125^I]PARPi-01, a radiolabeled olaparib derivative investigated in a triple-negative breast cancer model, showed rapid clearance, counterbalancing its effective radiotoxicity [219]. Improvements in that domain are therefore necessary before translation to the clinic.

### 5.6. Carbonic Anhydrase IX (CA IX) Inhibitors

Characterization of solid tumors is mostly conducted with hypoxia, eventually inducing genetic instability, subsequently leading to an aggressive tumor phenotype and treatment resistance [220]. Hypoxic cells use various mechanisms to adapt and survive in their environment. One of these mechanisms involves the expression of the carbonic anhydrase protein, which is part of a group of zinc metalloenzymes, playing a vital role in several physiological processes [221]. In particular, two isoforms of this protein, CA IX and CA XII, are associated with tumors. While the first is found in most solid tumors and gastrointestinal mucosa, the latter is present in not only tumors but also several other organs. Therefore, CA IX is a more suitable biomarker from a clinical perspective [222]. Additionally, the expression of CA IX is correlated with treatment resistance and with metastases, and is globally a marker of cancer’s poor prognosis.

CA IX is a protein with a molecular weight of 50 kDa and it consists of several domains including proteoglycan-like, catalytic, transmembrane, and intracellular domains. The catalytic domain of CA IX is located on the outer membrane of the cell and contains a zinc atom in its active center. Most CA inhibitors aim at targeting this extracellular catalytic domain. These entities contain a segment that can bind to zinc, either by attaching directly to the Zn^2+^ cation or indirectly by forming adducts with zinc-bound water or hydroxyl outside of the active site. The first group of entities comprises potent inhibitors of sulfonamide scaffold derivatives. The second group is composed of synthetic molecules, peptidomimetics, and antibodies [223]. Several radiolabeled inhibitors have been described, mostly for imaging [224]. Apart from radiolabeled antibodies, there has only been one small molecule reported so far for radionuclide therapy. This molecule is a radiocomplex called [^90^Y]Y-US2, which is labeled with yttrium-90 and contains a ureidosulfonamide group. Its efficacy has been tested in mice with CA IX tumors expressing high levels of the protein. [225]. In this study, mice that were administered [^90^Y]Y-US2 experienced a significant delay in tumor growth compared to the non-treated mice. Furthermore, limited hematological toxicity was observed. Although not as extensively studied as antibodies, small synthetic molecules have potential as radiotherapeutic drugs due to their superior pharmacokinetic properties and potentially lower toxicity. Further research is needed to explore their potential [226].

### 5.7. Vitamins

**Figure 9:**
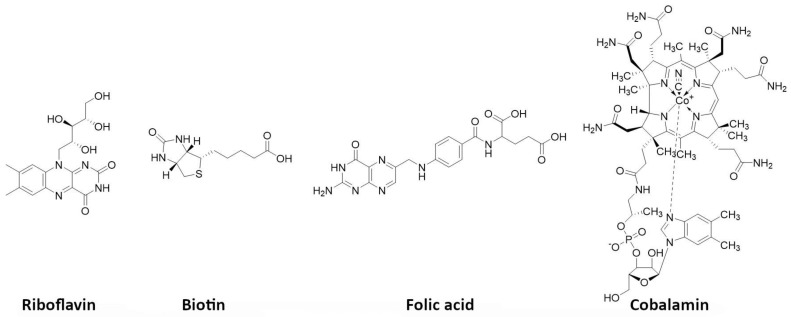
Vitamins of interest as receptor-specific targeting agents.

Vitamins are essential nutrients for the organism. In particular, they are critical for rapidly dividing cells, such as malignant cancer cells. B vitamins, including riboflavin (B2), biotin (B7), folic acid (B9), and cobalamins (B12), play a crucial role in tumor growth, with many solid tumor cells overexpressing receptors that facilitate their uptake (Figure 9). As a result, targeting these receptors has become an appealing approach. Numerous studies have shown that vitamin-based bioconjugates, particularly folate, have significant potential for imaging and therapy [227].

Biotin, a water-soluble vitamin, also referred as vitamin B7 or H, weighs 244 Daltons and plays a significant role in various metabolic processes, including gluconeogenesis and cell growth stimulation. The vitamin is internalized by binding to the sodium-dependent multivitamin transporter (SMVT) located on the surface of the cells. This transporter is present in high amounts in certain cancer types, such as colon, breast, lung, renal, and ovarian cancers. In such cancers, targeting with biotinylated agents might be an interesting strategy [228]. However, to date, apart from some imaging applications, radiolabeled biotin derivatives have only been applied as a tool for pretargeting, using the streptavidin/biotin approach [229].

Folic acid, also known as vitamin B9 or folacin, is a crucial nutrient that the body needs to make DNA and RNA and to metabolize amino acids required for cell division. Folate receptors (FRs) can internalize it, making it possible to selectively deliver a drug and facilitate its complete and rapid internalization by receptor endocytosis. Among all vitamins, folic acid is the most extensively investigated for tumor targeting [230]. FRs are 38–44 kDa glycoproteins that anchor in the cell membrane using a glycosylphosphatidylinositol domain, with limited expression in healthy tissues, except for the kidneys. In contrast, several solid epithelial tumors, including breast, cervical, colorectal, endometrial renal, nasopharyngeal, and ovarian carcinomas, as well as tumor-associated macrophages overexpress FRs. Targeting FRs has thus gained significant interest for developing imaging and therapeutic agents for these cancers [231,232]. Folate derivatives bind to FRs with high affinity and clear rapidly from FR-negative tissues. Hydrophilic folate conjugates present a better biodistribution profile than lipophilic ones, but kidney uptake is dose-limiting. This can be mitigated by using diuretics or pretreating with antifolate drugs [233,234]. Albumin-binding moieties can prolong blood circulation, heightening the tumor-to-kidney ratio and potentially making folate-based radiopharmaceuticals safe for clinical use [235]. Currently, one single radiolabeled FR-targeting agent has been investigated in clinic, for PET imaging. A ^177^Lu-radiolabeled one has been investigated in preclinical models and has been shown to significantly inhibit tumor growth without radiotoxicity. Substituting ^177^Lu with ^161^Tb increased the therapeutic efficacy [236,237].

Vitamin B12, also known as cobalamin, is crucial for the function of methionine synthase, an enzyme that controls one of the primary pathways for the production of folates. Its uptake into cells is made possible by the plasma carrier protein transcobalamin II (TCII), which is often highly expressed in many types of tumors. In cancer cells, TCII facilitates cell uptake by binding to upregulated specific receptors (i.e., CD320). Investigations have been conducted on various radiolabeled cobalamin derivatives, mostly using γ-emitters, both in preclinical models and in patients with different tumor types that express TCII [227]. One example of a positron-emitting cobalamin derivative (with ^89^Zr) has also been reported [238]. So far, the only known use of cobalamin in a therapeutic setting involves its application as a receptor-specific radiosensitizer in combination with external-beam radiotherapy. In a pancreatic adenocarcinoma preclinical model, cobalamin was used as a vector for a fluorophore (Bodipy650) [239].

### 5.8. Phospholipid Ether Analogues

Several years ago, phospholipid ether (PLE) analogues of the naturally occurring alkyl lysophospholipid and alkylphosphocholines have been demonstrated to be taken up preferentially by cancer cells and, moreover, to display antitumor and antimetastatic activities [240]. Combination with radiotherapy has shown interesting results [241]. However, the metabolic difference between neoplastic and normal cells could be exploited as a means to selectively target tumors and deliver a radioactive payload into the cancer cell. Indeed, PLE analogues accumulate in cancer cells, exploiting the altered lipid composition of tumor cell membranes. PLEs are taken up into cells via lipid rafts, cholesterol-rich regions of the plasma membrane, which have highly increased amounts in malignant cells as opposed to normal cells [242]. When the lipid rafts undergo transmembrane flipping, PLEs are internalized, delivering the radioactive payload intracellularly and, in the case of a therapeutic radionuclide, causing double-strand DNA breaks, eventually leading to apoptosis. Major advantage of this system is based on the fact it is broadly applicable to a wide variety of cancer types, indiscriminately targeting all cells within a tumor, independently of the expression of a specific antigen [243].

Radioiodinated PLE analogues were therefore developed, both for imaging and therapy of a variety of hematologic and solid tumor types [244,245,246,247]. Structure-activity studies demonstrated localization and clearance properties of these radiotracers depend on the length of the alkyl chain [248]. This eventually led to the development of ^131^I-radiolabeled iopofosine (18-(p-[^131^I] iodophenyl)octadecylphosphocholine), currently evaluated in several in the phase I and phase II trials [249,250,251,252,253,254,255]. A radiometallated analog (NM600) has been reported, labeled with ^86/90^Y and ^177^Lu, that preclinically demonstrated its ability to immunomodulate the TME in several tumor models, facilitating combined immunotherapy treatment [256,257].

### 5.9. Melanin Targeting Agents

Melanoma is a malignant disease originating from melanocytes. It is the deadliest and the most common skin cancer. A late diagnosis can drastically reduce survival rate, because of the ability of melanoma metastases to quickly disseminate. The search for a radiopharmaceutical with high affinity for melanoma, for early detection, and subsequent therapy, has therefore been an active field of investigations [258]. RIT, targeting surface antigens, and PRRT, targeting melanocortin receptors, hardly reached the clinical testing stage. In that context, targeting melanin appeared as particularly attractive [259]. The melanin pigment is an antigen present in >92% of melanomas [260]. Its primary function is to protect the skin against UV-induced damage, but because of increased tyrosinase activity, it is overexpressed in melanoma cells. It is expressed intracellularly, but thanks to the high cellular turnover of tumor cells, some melanin can be found at the extracellular level, and thus be easily accessible for targeting. Some therapeutic melanin-binding antibodies have been successfully developed, notably labeled with rhenium-188 [261]. However, most promising results have been obtained with small molecules, which are able to bind to intracellular melanin with high specificity.

**Figure 10:**
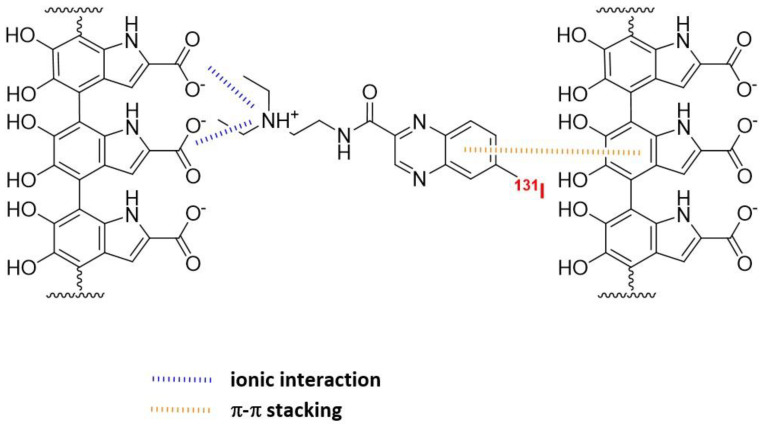
Proposed binding mechanism of a benzamide derivative ([^131^I]ICF01012) with melanin fragment (adapted from [251]).

Organic compounds having aromatic rings and a tertiary amino group have been demonstrated to exhibit high affinity for melanin. These include dyes, such as methylene blue or acridine orange, and benzamide or heteroarylcarboxamide derivatives or related compounds [262]. It is hypothesized that these compounds strongly bind to melanin fragments, both through π-interaction between aromatic rings of the radiotracer and indole moieties of melanin and ionic interaction between the protonated cation of the tertiary amine and melanin’s carboxylates (Figure 10). Several benzamide derivatives have been synthesized and evaluated to find the compound with the best characteristics for melanoma uptake [263,264,265]. Even though some derivatives have been labeled with metals, radioiodinated compounds appeared as the most attractive ones [265,266].

Several of these compounds, radiolabeled with iodine-123, have been investigated in small clinical trials. [^123^I]BZA (*N*-(2-diethylaminoethyl)-4-iodobenzamide) has even been evaluated in a phase 2 trial, including 110 patients. It demonstrated 81% sensitivity and 100% specificity [267]. On the contrary, most of the therapeutic derivatives, labeled with iodine-131 or astatine-211, are still in a preclinical stage, with promising outcomes [268,269,270,271]. Some of these radiotracers have nonetheless been evaluated in patients. In a pilot study including 26 patients with histologically proven metastasized malignant melanoma, [^131^I]I-BA52 (*N*-(4-((2-diethylaminoethylcarbamoyl)-2-iodo-5-methoxyphenyl)benzo [1,3]dioxole-5-carboxamide) was effective in three of the five patients who were treated with a single dose over 4.3 GBq [272]. More recently, [^131^I]ICF01012 (*N*-(2-diethylaminoethyl)-6-iodoquinoxaline-2-carb oxamide) is currently being evaluated in a dose-escalation phase 1 trial [273]. Though [^211^At]-methylene blue was alleged to have been granted approval for a phase 1 clinical trial, no subsequent clinical trial seems to have been initiated [268].

## 6. Conclusions

Targeted radionuclide therapy has evolved towards precision medicine, especially when used with a companion diagnostic agent, to enhance patient selection and monitoring of treatment response. The understanding of the inter- and intra-individual heterogeneity of tumors and advances in radiobiology have led to the improvement of the theranostic approach, which now appears as essential in patient’s and disease’s management. The ALSYMPCA, NETTER-1, and VISION phase III trials have demonstrated the effectiveness of targeted radionuclide therapy in treating bone metastases of mCRPC patients, neuroendocrine tumors, and prostate cancer, respectively, thereby inspiring further research. The latest research has shown a shift towards antagonists and α-emitters, as well as a pan-cancer approach that targets more widely expressed components of the tumor microenvironment, such as CXCR4 and FAPI radioconjugates. However, the challenge is now to select the most suitable agent, with the most suitable nuclide, for each patient. Carefully designed clinical trials are now compulsory to explore the dosimetry, new indications, and combination therapies of promising agents. The focus is also shifting from treating bulky tumors to treating minimal residual malignancies, where targeted radionuclide therapy is most appropriate. As pharmaceutical companies show increasing interest, targeted radionuclide therapy is set to become an essential component of nuclear medicine and, to a greater extent, of oncology.

## Figures and Tables

**Table 1:** Main radionuclides used in therapy.

Radionuclide	Half-life	Energy (MeV)	E_γ_ (keV)	Tissue Penetration Range (mm)
β-emitter				
^90^Y	2.7 days	2.284	/	12
^131^I	8 days	0.81	0.364	2.4
^161^Tb	6.9 days	0.593	74.6	3
^177^Lu	6.7 days	0.497	208113	2.2
^188^Re	17 h	2.118	155	11
α-emitter				
^149^Tb	4.1 h	3.97	Multiple emissions (165–800)	<100 µm
^211^At	7.2 h	7.45	85 (X-ray)	
^212^Pb/^212^Bi *	10.6 h	8.78	238, 300	
^213^Bi	0.8 h	8.38	440	
^223^Ra	11.4 days	5.71, 6.82, 7.39, 6.62	270	
^225^Ac	10 days	5.8, 6.3, 7.1, 8.38	218, 440 (from daughters)	
^227^Th	18.7 days	6.14, 5.71, 6.82, 7.39, 6.62	236	
Auger e^−^ emitter				
^111^In	2.8 days	0.007	405	<1 µm
^125^I	60 days	0.019	42	

* ^212^Pb is a β^−^-emitter, but is usually considered for TAT, acting as an in vivo generator for α-emitting ^212^Bi [9].

**Table 2:** Peptides suitable for tumor targeting: their receptors (in bold, receptor types overexpressed in human) and tumor expression.

Peptide	Receptor	Tumor Expression
α-Melanocyte-stimulating hormone	**MCR_1_**, MCR_3_, and MCR_5_	Melanomas
Bombesin/Gastrin-releasing peptide	BB_1_, **BB_2_ (GRPR)**, BB_3_, and BB_4_	Glioblastomas, prostate, breast, pancreatic, gastric, colorectal cancers, and small cell lung
Cholecystokinin/gastrin	CCK_1_, and **CCK_2_**	Adenomas, astrocytomas, gastrointestinal and ovarian stromal tumors, medullary thyroid, pancreatic and small cell lung cancers
Epidermal Growth Factor	**EGFR**	Breast cancer
Exendin	**GLP-1**	Gastrinomas, insulinomas, medullary thyroid carcinomas, paragangliomas and pheochromocytomas
Gonadotropin-releasing hormone	**GnRH-R**	Breast and prostate cancers
Neuropeptide Y	**Y1**, Y2, Y4, and Y5	Breast, ovary, adrenal, brain, kidney, GI-tract, and bone (Ewing’s sarcoma)
Neurotensin	**NTR_1_**, NTR_2_, and NTR_3_	Breast, colon, pancreatic, prostate, small cell lung cancers, and meningiomas
RGD	**α_V_β_3_ integrin**	Tumor-induced angiogenesis
SDF-1α/CXCL12	**CXCR4**, and CXCR7	Leukemias, lymphomas, melanomas, brain, breast, kidney, lung, ovarian, pancreas, and prostate tumors
Somatostatin	Sstr1, **sstr2**, sstr3, sstr4, and sstr5	Neuroendocrine tumors, lymphomas, paragangliomas, brain, breast, renal, and small cell lung cancers
Substance P	**NK_1_**, NK_2_, and NK_3_	Glial tumors, breast, medullary thyroid, pancreas, and small cell lung cancers
Vasoactive intestinal peptide	**VPAC_1_**, and VPAC_2_	Bladder, breast, gastrointestinal, non-small cell lung, ovarian, pancreatic, and prostate cancers

**Table 3:** Peptidic sequences of mainly clinical used somatostatin agonist analogs.

Peptide	Peptidic Sequence
OC Octreotide	d-Phe-cyclo(Cys-Phe-d-Trp-Lys-Thr-Cys)Thr(ol)
LAN Lanreotide	β-d-Nal-cyclo(Cys-Tyr-d-Trp-Lys-Val-Cys)Thr-NH_2_
VAP Vapreotide	d-Phe-cyclo(Cys-Phe-d-Trp-Lys-Val-Cys)Trp-NH_2_
TOC [Tyr^3^]-Octreotide	d-Phe-cyclo(Cys-Tyr-d-Trp-Lys-Thr-Cys)Thr(ol)
TATE [Tyr^3^]-Octreotate	d-Phe-cyclo(Cys-Tyr-d-Trp-Lys-Thr-Cys)Thr
NOC [1-Nal^3^]-Octreotide	d-Phe-cyclo(Cys-1-Nal-d-Trp-Lys-Thr-Cys)Thr(ol)
SOM230 Pasireotide	Cyclo(Hyp(Unk)-Phg- d-Trp-Lys-Tyr(Bn)-Phe)
P2045 Tozaride	Ser-Thr-Cys(Trt)-Phe(4-NH_2_)-(β-DAP)-CH_2_CO-S-cyclo((N-Me)HCy-Phe-Tyr-d-Trp-Lys-Thr)

**Table 4:** Main antagonists currently investigated.

Target	Name	Structure
SSTR	DOTA-BASS	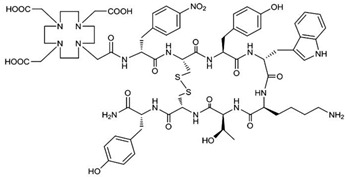
	DOTA-LM3	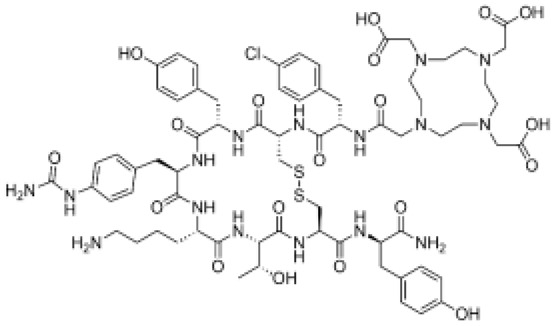
	DOTA-JR11 (Satoreotide)	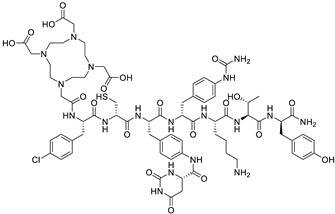
GRPR	DOTA-RM2	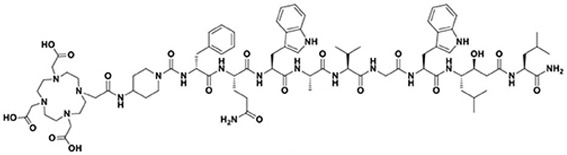
	NeoB	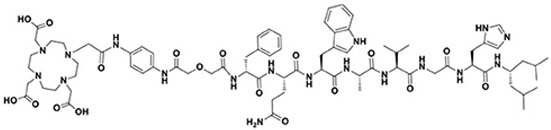
NTR	3BP-227	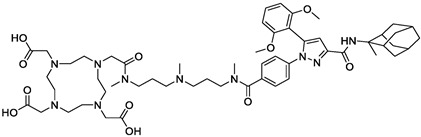

**Table 5:** Radiotherapeutic bone-seeking agents.

Radionuclide	Agent
**Approved Agents for Clinical Use**	
Strontium-89 (β^−^) (50.5 d)	[^89^Sr]SrCl_2_—Metastron^®^
Samarium-153 (β^−^) (1.9 d)	EDTMP—Quadramet^®^
Rhenium-186 (β^−^) (3.7 d)	HEDP
Radium-223 (α) (11.4 d)	[^223^Ra]RaCl_2_—Xofigo^®^
**Agents in Clinical Trials**	
Rhenium-188 (β^−^) (17 h)	HEDP Zoledronic acid
Lutetium-177 (β^−^) (6.8 d)	EDTMP DOTMP * Zoledronic acid (Dotazol)
Holmium-166 (β^−^) (1.1 d)	DOTMP *
Tin-117m (CE) (13.6 d)	DTPA **

* DOTMP = 1,4,7,10 tetraazacyclododecanetetramethylenephosponic acid. ** DTPA = diethylenetriaminepentaacetic acid.

## Data Availability

Not applicable.
